# Voltammetric Determination of Amoxicillin Using a Reduced Graphite Oxide Nanosheet Electrode

**DOI:** 10.1155/2021/8823452

**Published:** 2021-04-28

**Authors:** Thi Hai Yen Pham, Thi Trang Mai, Hoang Anh Nguyen, Thi Thu Hien Chu, Thi Thu Ha Vu, Quoc Hung Le

**Affiliations:** ^1^Institute of Chemistry, Vietnam Academy of Science Technology, 18 Hoang Quoc Viet, Cau Giay, Hanoi 100000, Vietnam; ^2^VNU University of Science Hanoi, 19 Le Thanh Tong, Hoan Kiem, Hanoi 100000, Vietnam; ^3^Department of Chemistry, National University of Civil Engineering (NUCE), 55, Giai Phong, Hai Ba Trung, Hanoi 100000, Vietnam; ^4^University of Science and Technology of Hanoi (USTH), Vietnam Academy of Science and Technology, 18 Hoang Quoc Viet, Cau Giay, Hanoi 100000, Vietnam

## Abstract

A reduced graphite oxide nanosheet electrode (RGOnS) was prepared as a sensor for amoxicillin (AMX) detection, an antibiotic commonly used in the livestock farm, by the square-wave adsorptive stripping voltammetry technique. Graphite oxide with nanosheet shape was produced from a graphite electrode by a chronoamperometry process at 5 V and then an electrochemical reduction process was carried out to form RGOnS with restored long-range conjugated networks and better conductivity. The electrodes were characterized by SEM, EDX, and FTIR spectroscopy. The RGOnS electrode prepared at an optimal reduction potential of −1 V for 120 s exhibits a larger electrochemical active surface area, and the obtained oxidation signal of AMX is approximately ten times higher than that of the pristine graphite electrode. The analytical conditions such as the pH of electrolyte and accumulation time were optimized. The calibration curve built under the optimal conditions provided a good linear relationship in the range of AMX concentration from 0.5–80 *µ*M with the correlation coefficient of 0.9992. The limit of detection was calculated as 0.193 *µ*M. Satisfactory results are obtained from the detection of the AMX in different samples using the prepared electrode.

## 1. Introduction

Since the first discovery in Beecham Research Laboratories in 1972, amoxicillin, one of the penicillin-derived antibiotics, has been universally used to treat common infectious diseases in humans such as otitis, pneumonia, and genital diseases [1–3]. This kind of antimicrobial drug is also employed in food animal production to prevent infections and boost the growth of animals [4–6]. Accordingly, amoxicillin can be easily released to the environment, such as water body from human excretion, wastewater from food animal production, and hospital wastewater discharges [7–9] due to incomplete waste water treatment. Despite the crucial role of this antibacterial drug, amoxicillin is considered a harmful substance to the ecosystem when it is introduced to the environment. Like other antibiotic substances, the contamination of amoxicillin in the environment, even at a low level, gives rise to the growth of antibiotic-resistant bacteria, which leads to potential risk to human life and livestock [4, 10–12]. Therefore, it is necessary to establish rapid and sensitive methods for the monitoring of amoxicillin at the trace level in the aquatic environment. Apart from common advanced methods for amoxicillin detection such as high-performance liquid chromatography (HPLC) with fluorescence detector, HPLC with ultraviolet-visible detector, chemiluminescence, high-performance liquid chromatography-tandem mass spectrometry, and ultra performance liquid chromatography-mass spectrometry [13–16]; electrochemical methods have been widely applied in the analysis of antibiotics in real samples in the recent decades because of its advantages such as low cost, simple operation, fast measurement, and good sensitivity and selectivity [17]. Additionally, electrochemical methods can be easily performed for quick analysis of contaminants on-site [18].

In the electrochemical method, working electrodes play a significant role in the analytical measurement because the reduction-oxidation reaction of analytes takes place on the electrode surface. Therefore, scientists have attempted to modify the electrode by advanced methods with the aim of enhancing the analyte signals. Recently, numerous studies have applied nanocarbon-based materials (carbon nanotubes, graphene, graphene oxide) to electrode modification as medical, biological sensors because of their unique physical-chemical properties such as a large electroactive surface area, excellent electron transfer, good electrocatalytic properties, and high chemical resistance [17, 19]. Carbon nanotubes were reported as the electrode material for the detection of biological substances such as antibiotics, glucose, ascorbic acid, dopamine, and uric acid [20–23]. Moreover, the combination of graphene with other metals, metal oxides, or polymers has also been employed in the quantification of some antibiotics such as cloxacillin and chloramphenicol antibiotic in milk samples [24, 25].

Amoxicillin is an electroactive species and can therefore be quantified by electrochemical methods. The electrochemical oxidation of AMX was proposed by some authors [26, 27], where the -OH group attached to the aromatic ring donates one electron to form the >C=O group. Due to the advantages of nanocarbon mentioned above, various applications of nanocarbon-based materials in electrode modification to improve the sensitivity of AMX detection were reported. In 2009, *Behzad Rezaei* and *Sajjad Damiri* modified glassy carbon substrate by multiwall carbon nanotubes to detect amoxicillin in pharmaceutical and human urine samples [28]. The composite material of carbon nanotubes and gold nanoparticles was employed in the determination of amoxicillin in bovine milk by *Muhammad* et al. [29]. Graphene and graphene oxide decorated by metal nanoparticles such as gold, palladium, and copper were investigated in some previous studies for the enhancement of amoxicillin electrochemical signals [30, 31]. In addition, many organic substances containing functional groups that increased the binding ability with AMX were used to modify electrode surfaces along with nanocarbon. *Deroco* et al. focused on the modification of the glassy carbon electrode (GCE) by carbon black attached to the di-hexadecyl phosphate membrane [32]. In *Deroco*'s study, when the unmodified GCE was used, no AMX oxidation process was observed; however, a well-defined oxidation peak for AMX was obtained using the modified electrode due to its electrochemical catalytic activity and strong interaction with AMX.

In many studies [28, 29, 32–37], the nanocarbon-modified electrodes were prepared by dip-coating or drop-casting methods. These kinds of modifications can lead to unstable bonding between modifiers and the substrate and, therefore, restrict the electron transfer in the redox reaction and the signal of desirable analytes. In this study, with the purpose to improve the electron transfer and the stability, reduced graphite oxide nanosheets were produced directly by the electrochemical expansion of the graphite electrode surface using a rapid, simple, and green electrochemical method. The modified electrode was first applied as an effective electrochemical sensor for amoxicillin detection.

## 2. Experiments

### 2.1. Reagents and Apparatus

#### 2.1.1. Reagents

Amoxicillin trihydrate (C_16_H_19_N_3_O_5_S·3H_2_O, 98.0%) was purchased from bioWorld, USA. H_2_SO_4_ (98.0%), H_3_PO_4_ (85%), KOH (≥85.0%), KH_2_PO_4_ (≥99.5%), and K_2_HPO_4_ (≥98.0%) were supplied by Merck (Germany), and K_3_[Fe(CN)_6_] (≥99.0%) was obtained from Sigma-Aldrich. All chemicals and reagents were of analytical grade and used without further purification.

A 0.5 M H_2_SO_4_ solution used as a reagent for graphite surface expansion and reduction of the graphite oxide nanosheet electrode was diluted from 98% H_2_SO_4_ with double distilled water. Phosphate buffer solutions (PBS) with different pH values from 6.0 to 10.0 were prepared from 0.2 M KH_2_PO_4_, K_2_HPO_4_ in double distilled water, and the pH was adjusted by adding 1.0 M KOH or 1.0 M H_3_PO_4_ solutions. The buffer solutions were used as a supporting electrolyte in amoxicillin (AMX) detection.

0.5 mM and 5 mM AMX stock solutions were prepared by dissolving AMX powder in double distilled water. Standard solutions were prepared fresh daily just before use by dilution of the stock solution in PBS.

#### 2.1.2. Apparatus

The surface of the electrodes was examined using field emission scanning electron microscopy (FESEM) (Hitachi S-4800; Japan) at different magnifications and energy dispersive X-ray (EDX) spectra (Horiba 7593-H; England). Fourier transform infrared spectra (FTIR) were obtained on a Spectrum Two FTIR spectrometer (PerkinElmer 102717; UK).

Electrochemical measurements were performed with a custom-made multifunctional potentiogalvanostat manufactured in the Institute of Chemistry, Vietnam Academy of Science and Technology, Hanoi, Vietnam. The electrochemical system was established with three electrodes, where the platinum wire and Ag/AgCl electrode were used as a counter electrode and a reference electrode, respectively; the working electrodes were fabricated electrodes such as graphite electrode (GE), graphite oxide nanosheet electrode (GOnS), and reduced graphite oxide nanosheet electrode (RGOnS). Electrochemical characteristics of electrodes were investigated by cyclic voltammetry (CV) in 0.1 M PBS at pH 7.0 containing 5 mM K_3_[Fe(CN)_6_] and in the analyte. The ability of fabricated electrodes for AMX detection was explored by the square-wave adsorptive stripping voltammetry (SWAdSV) technique in PBS solutions containing AMX.

### 2.2. Preparation and Characterization of Graphite Oxide Nanosheet Electrode (GOnS) and Reduced Graphite Oxide Nanosheet Electrode (RGOnS)

The disk graphite electrode (99.9% graphite, diameter of 3.5 mm; Japan) was polished with 3000 and 5000 grit sandpaper to get a flat and smooth surface and rinsed with double distilled water. Then, the ultrasonication of the electrode in ethanol 90% was carried out to eliminate potential contaminants. After rinsing with double distilled water, the electrode surface was electrochemically expanded by the potentiostatic technique at 5 V potential for 1 s in 0.5 M H_2_SO_4_ solution to obtain a graphite oxide nanosheet electrode. After that, the expanded electrode was reduced by applying a constant potential for 120 s to form a reduced graphite oxide nanosheet electrode named RGOnS.

### 2.3. Preparation of Real Samples

Distilled water, tap water, West Lake water, and domestic wastewater samples were collected and stored at 4°C. After filtration, the samples were spiked with a known amount of AMX. To prepare the drug sample solution, the drug powder containing 500 mg AMX in a capsule was finely ground in an agate mortar, then extracted in methanol/water (1 : 1, v/v) solution under the ultrasonic condition for 30 min. After filtration to remove insoluble substances, the extract obtained was made up to 500 mL in a volumetric flask to obtain a drug sample solution. The AMX concentration in spiked samples and the drug sample solution was determined by the standard addition method.

All experiments in this study were performed at room temperature (25 ± 1°C).

## 3. Results and Discussion

### 3.1. Surface Properties of Graphite Oxide Nanosheet Electrode and Reduced Graphite Oxide Nanosheet Electrode

The surface morphologies of the graphite and graphite oxide nanosheet electrodes were characterized by SEM.

**Figure 1:**
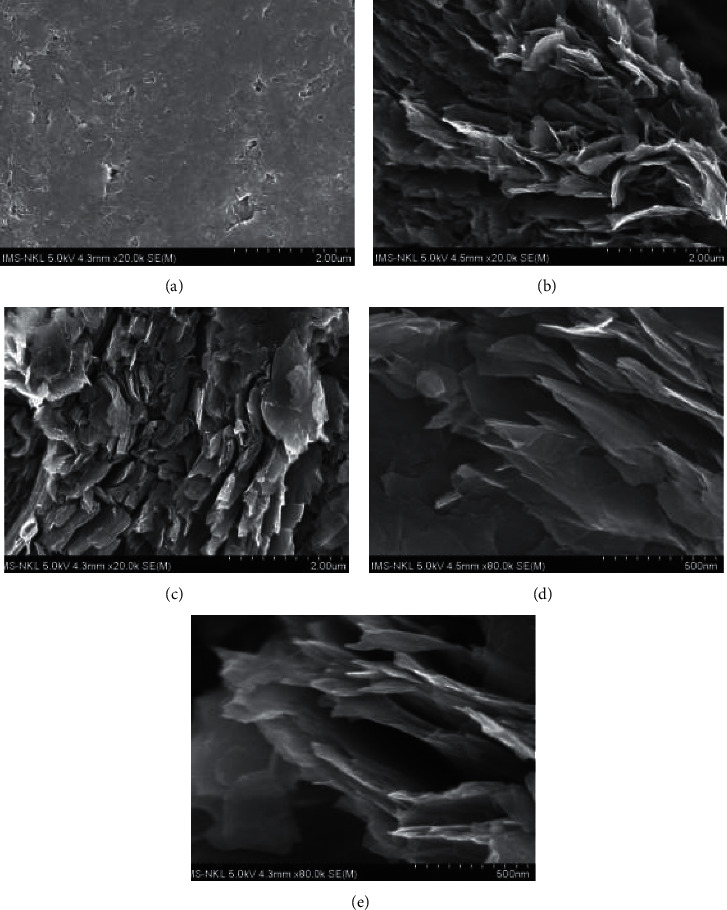
SEM images of graphite electrode (a), graphite oxide nanosheet electrode (b, d), and reduced graphite oxide nanosheet electrode (c, e) at different magnifications.

Figure 1(a) shows a smooth and homogenous surface of the graphite electrode. In this image, scattered narrow cracks between graphite layers are clearly observed that exhibits a tight stacking of layers as a feature of the graphite structure. When the graphite electrode was oxidized by applying a potential of 5 V in 0.5 M H_2_SO_4_ solution to produce GOnS, graphite layers expanded and isolated to form graphite oxide nanosheets on the surface electrode. The obtained GOnS surface is well observed with individual flakes consisting of multiple nanosheet structure (Figures 1(b) and 1(d)). Under the magnification of 80.0k (Figure 1(d)), the extremely thin shape of graphite oxide sheets with a few nanometers thick are clearly seen; and the distance between flakes is estimated to be from tens to several hundreds of nanometers. After the electrochemical reduction of GOnS to form RGOnS, the multiple nanosheet structure still remained on the electrode surface that is exhibited by the similar morphology in the SEM images of RGOnS (Figure 1(c) and 1(e)) to GOnS in the same magnifications. The great formation of multiple nanosheet structure could cause a significant increase in the specific surface area of modified electrodes compared with to the original one, which would be beneficial for the enhancement of analyte electrochemical signals.

The mechanism of expansion was proposed by *Parvez* in 2013 and 2014, and *Yu* in 2015. According to the studies, the electrolysis of water at the high applied voltage produces hydroxyl ions (OH^−^) at the edge sites and grain boundaries of the graphite electrode. The interaction of two vicinal -OH groups can form epoxide rings or be oxidized to create carbonyl groups. Their formation expands the graphite layers leading to the intercalation of H_2_O, SO_4_^2−^ into the lattice network. Then, the gaseous O_2_ and CO_2_ generation inside the lattice network through the oxidation reaction efficiently support the expansion of graphite nanolayers [38–40].

**Figure 2:**
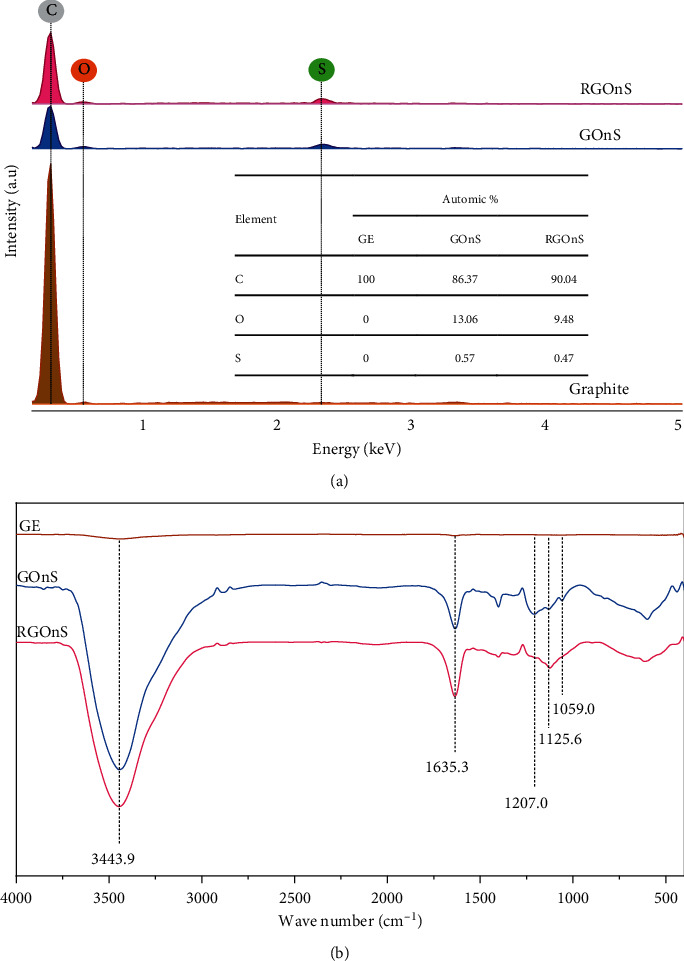
EDX (a) and FTIR (b) spectra of graphite, graphite oxide nanosheet electrode (GOnS), and reduced graphite oxide nanosheet electrode (RGOnS).

The element percentage at the electrode surface was estimated from EDX spectra, as shown in Figure 2. While the graphite electrode contained 100% carbon, the EDX spectra of both GOnS and RGOnS indicate the presence of oxygen in the composition after the oxidation of GE in a 0.5 M H_2_SO_4_ solution, which was attributed to the formation of oxygen functional groups. The proportion of oxygen existing on the surface of GOnS was up to 13.06%. After undergoing a reduction at −1 V for 120 s, the percentage of oxygen considerably decreased by nearly one-third to 9.48%. As observed in the spectra, a sulfur element was present in a small proportion on GOnS and RGOnS electrode surfaces, which is due to the intercalation of sulfate ions (SO_4_^2−^) within graphitic layers during the expansion. After the process, a small amount of sulfate ions still retained or C–S bonds were formed on the electrode surface. This phenomenon is in agreement with previous studies [41, 42].

The FTIR spectra of the electrode surface were used to investigate the change in the chemical structure before and after the electrochemical modification.  Figure 2(b) depicts the FTIR spectra of GE, GOnS, and RGOnS. As observed, the peaks of unmodified graphite are not clearly seen; while in GOnS and RGOnS spectra, some typical peaks are well defined with noticeably high intensities. The peak at 3443.9 cm^−1^ attributed to O–H stretching vibration dramatically increased after the GE oxidation, which reveals the remarkable formation of C–O–H groups on the GOnS surface [43, 44]. The same peak was also acquired in the RGOnS spectrum, but the peak intensity was slightly lower because an amount of C–O–H group could be electrochemically reduced to C–C or C=C. This restoration of C=C bonding on RGOnS was confirmed by a slight increase in peak intensity at 1635 cm^−1^ (C=C stretching) compared with that of GOnS. The peaks at 1207 cm^−1^ and 1059 cm^−1^ appeared on the GOnS spectrum referring to the stretching of C–O in epoxy and alkoxy groups [45], while these peaks mostly disappeared on the RGOnS spectrum after the reduction of GOnS. The FTIR spectra prove the successful reduction of GOnS to restore C=C bonding (*π* electron conjunction) in the lattice network of RGOnS that facilitated the electron transfer in the AMX oxidation in further experiments.

### 3.2. Electrochemical Properties of GOnS and RGOnS Electrodes

**Figure 3:**
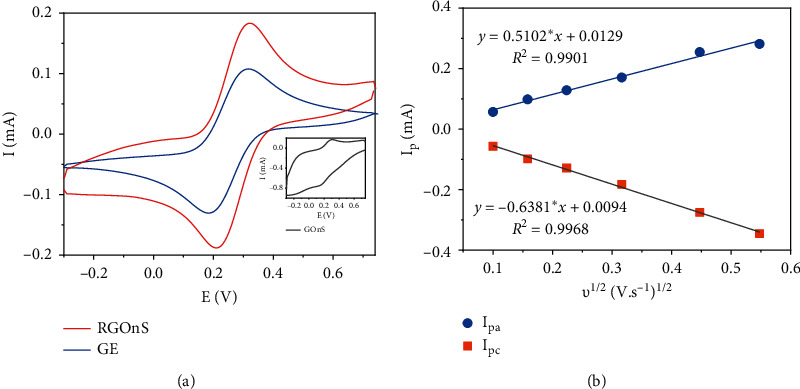
Cyclic voltammograms of graphite, reduced graphite oxide nanosheet electrodes, and graphite oxide nanosheet (inset) in 0.1 M PBS (pH 7.0) containing 5 mM K_3_[Fe(CN)_6_] at *v*  = 100 mV/s (a) and the dependence of cathode and anode peak currents (I_pa_ and I_pc_) on the square root of scan rate on RGOnS (b).

Electrochemical properties of modified electrodes (GOnS and RGOnS) were evaluated by cyclic voltammetry of redox probe Fe(CN)_6_^3−/4−^, which is shown in Figure 3. It is notable that reversible cyclic voltammograms (CVs) were acquired when using graphite and RGOnS electrodes with I_pc_/I_pa_ ≈ 1 (Figure 3(a)). The reversible characteristic of the redox probe Fe(CN)_6_^3−/4−^ on RGOnS was also displayed through the linear increase of peak height with the increase of the square root of scan rate (*v*^1/2^) in both cathode and anode reactions (*R*^2^_cathode_ = 0.9968, *R*^2^_anode_ = 0.9901) (Figure 3(b)). Meanwhile, CVs of GOnS (Figure 3(a) inset) show a high reduction current attributed to the simultaneous reductions of Fe^3+^ and the oxidants formed on the GOnS surface after the expansion. The overlapping of these reactions made the reduction peak of Fe^3+^ indistinct.

Due to the above characteristics, the electrochemical reaction can be considered a diffusion-controlled process; thus, the electrochemical redox reaction of Fe(CN)_6_^3−/4−^ couple obeys the Randles–Sevcik equation, and the electrochemical active surface area (ECSA) of these electrodes was calculated via this equation [46, 47] based on the reduction peak of (FeCN)_6_^3−^. The calculated ECSA for the RGOnS electrode is approximately 1.3 times higher than that for the graphite electrode.

Figure 3(a) indicates that there were an increase in the peak height and a decrease in the difference of peak potentials (ΔE_p_) observed in the CV of the RGOnS electrode in comparison with the original graphite electrode. These changes were resulted from the higher electrochemical active surface area as well as the faster electron transfer on the RGOnS electrode. The growth of ECSA can be explained by the formation of nanosheet on the electrode surface. Obviously, each nanosheet has similar properties as reduced graphene oxide, which promotes the electron transfer process.

### 3.3. Electrochemical Behavior of AMX on the Prepared Electrode

**Figure 4:**
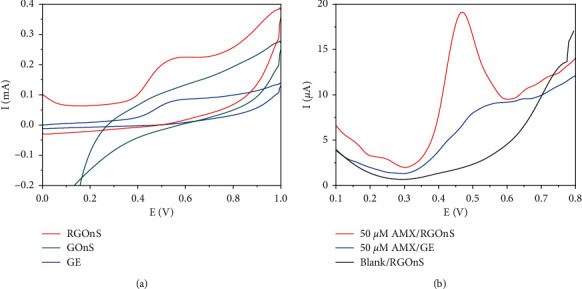
Cyclic voltammograms in 500 µM AMX solution at a scan rate of 50 mV/s (a) and square wave voltammograms in 50 µM AMX solution with 120 s accumulation (b) of studied electrodes. The electrolyte is 0.1 M PBS of pH 7.

The electrochemical behavior of AMX on graphite, GOnS, and RGOnS electrodes was investigated through their cyclic voltammograms (CVs) and square wave voltammograms (SWVs) as shown in Figure 4.

As can be seen in Figure 4(a) that in the PBS containing 500 µM AMX, well-defined peaks appeared at 0.52 V in the forward scan of CVs of both graphite and RGOnS electrodes. These peaks correspond to the electrochemical oxidation of AMX on the electrode surface. The nonobservation of the reduction peak at reverse scan indicates that the electrochemical behavior of amoxicillin on these electrodes is irreversible. Meanwhile, the GOnS electrode's CV exhibited an unclear peak. This can be explained by the low electron transfer and high charge-transfer resistance that were caused by the high content of oxygen-containing functional groups on the graphite oxide surface, as confirmed by FTIR [48, 49]. After GOnS underwent a reduction process to form RGOnS, the oxygen-group content was eliminated, and defects on GOnS were repaired to restore the long-range conjugated network. Thus, the conductivity of RGOnS was improved that promoted the electron transfer of the AMX oxidation on the electrode surface. However, RGOnS was not free of oxygen-containing functional groups such as -COOH and -OH on the reduced graphite sheets' edges that would facilitate the bonding with the analytes to induce the considerable enhancement of analytical signals [50–53]. The electrode surface improvement contributed to the significant increase in AMX signals. In particular, the SWV peak intensity of AMX on RGOnS after 120-s accumulation time of AMX is 9.7 times higher than that on the original graphite electrode (Figure 4(b)). Besides, the modified electrode also shows high electrocatalytic activity through the decrease of AMX overpotential in comparison with the original graphite electrode as seen in Figure 4(b).

### 3.4. Influence of GOnS Reduction Potential on the Signal of AMX

Regarding the reconstruction of the conjugated network in the RGOnS surface structure that affects the signal of AMX, the applied potential to reduce GOnS was investigated. In this experiment, the reduction potential (E_red_) values ranging from −0.2 V to −1.5 V were examined for 120 s.

**Figure 5:**
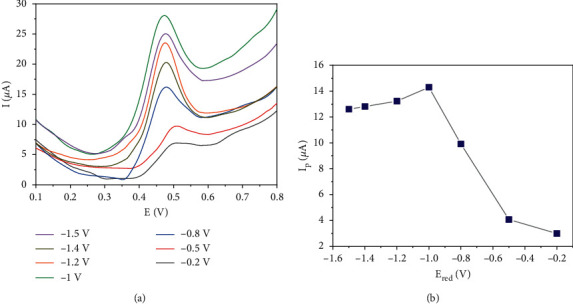
SWVs of RGOnS electrodes prepared at different E_red_ in PBS containing 50 *µ*M AMX (a) and plot of the AMX peak current (*I*_p_) vs. E_red_ (b).

As illustrated in Figure 5(a) and 5(b), when the applied potentials varied from −0.2 to −1 V, better peak currents of AMX were acquired. This can be explained by the better repair of the conjugated network on the RGOnS surface due to more reduction of Csp^3^ to Csp^2^ state at more negative reduction potentials, which facilitated the electron exchange and transfer in the network leading to the increase in the oxidation signal of AMX. The peak current reached the highest value as E_red_ = −1 V, and then it gradually decreased as E_red_ was changed from −1 V to −1.5 V. When the applied voltage was more negative than −1 V, the strong generation of H_2_ gas on the electrode surface occurred parallel to the reduction of oxygen-containing functional groups. This is believed to compete and restrict the reduction of Csp^3^ to Csp^2^. As a result, the regeneration of the *π*-conjugated network on the nanosheet at those reduction potentials was not efficient as at E_red_ of −1 V. Besides, with such negative potentials (from −1.2 to −1.5 V), the functional groups that facilitate the adsorption of the analyte may not be maintained in appropriate content. Therefore, −1 V potential was chosen for GOnS reduction performance to get the highest signal of AMX.

### 3.5. Optimization of Analysis Conditions

#### 3.5.1. Influence of electrolyte pH on the signal of AMX

**Scheme 1:**
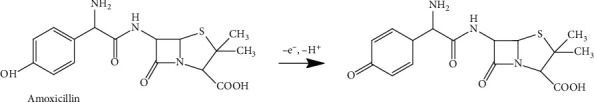
The Electrochemical oxidation process of amoxicillin.

**Figure 6:**
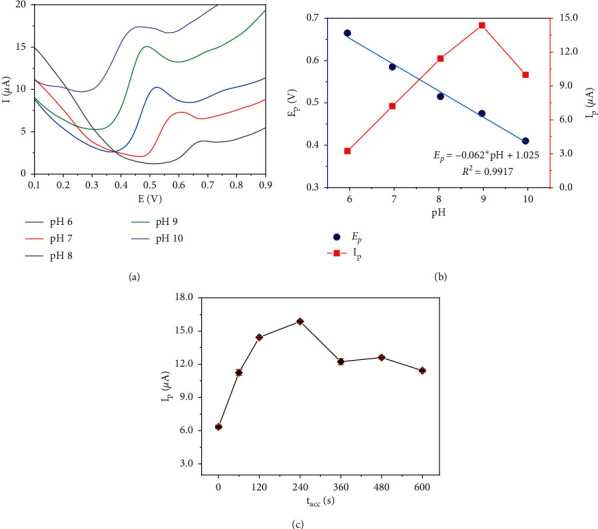
SWVs of RGOnS (with *E*_red_ = -1 V in PBS containing 50 *µ*M AMX at different pH (a) and plot of the AMX peak potential and peak current vs. pH (b). Plot of AMX peak current vs. t_acc_ (c).

Figure 6(a) and 6(b) represents the influence of electrolyte pH in the range from 6.0 to 10.0 on the SWV signal of 50 *µ*M AMX in 0.1 M PBS. It is obvious that the AMX oxidation peak potential (*E*_p_) shifted negatively with the increase of pH values, and a good linear relationship between these two parameters was shown in Figure 6(b) (blue color) with the linear regression equation: *E*_p_ = 1.025–0.062 pH (*R*^2^ = 0.9917). This result exhibits that the electrochemical oxidation of AMX was accompanied by proton transfer. The slope of −0.062 V/pH is close to the theoretical value of −0.059 V/pH given by the Nernstian equation with *n* = 1 that indicates the equal number of protons and electrons transfer process [54, 55]. This result is in consonance with previous studies [26, 27, 30, 56]. Accordingly, the electrochemical oxidation of AMX follows the mechanism as presented in Scheme 1.

As shown in Figure 6(b), the red line reveals that the peak intensity gradually increased with the rise of pH values; and the highest value was obtained at pH 9.0. Since the pH was adjusted to 10.0, the peak height experienced a significant decline. Thus, pH 9.0 was used as the optimal pH for the next experiment.

#### 3.5.2. Influence of accumulation time on the signal of AMX

The accumulation time (*t*_acc_) of AMX on the electrode surface has a significant impact on the SWV signals of AMX. The *t*_acc_ was examined in the range of 0 s–600 s to acquire an appropriate time for the analytical process. The result in Figure 6(c) shows a gradual growth of AMX peak current when *t*_acc_ changed from 0 s to 240 s that indicated the rapid adsorption of AMX onto the electrode surface. With longer *t*_acc_ from 240 s to 600 s, the AMX peak current experienced a decrease that attributed to the desorption of AMX on the electrode surface. Therefore, for further study, *t*_acc_ of 120 s was chosen as the adequate time to achieve wide linearity, high sensitivity, and time-saving.

### 3.6. Reproducibility of the RGOnS Electrode

**Figure 7:**
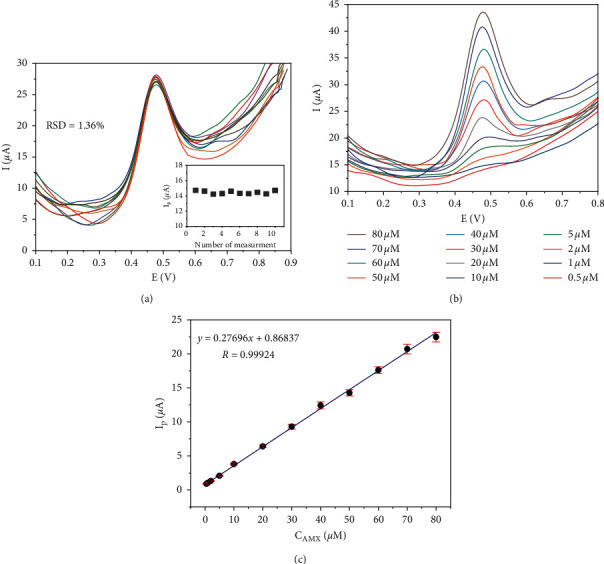
SWVs obtained on 10 different RGOnS electrodes at 50 *µ*M AMX (a), inset is AMX peak height in 10 measurements. The SWVs of AMX with concentration from 0.5 to 80 *µ*M (b) and calibration curve (c).

The reproducibility of the electrode plays a crucial role in the analysis because there was a considerable decrease in the analyte signals after successive measurements, resulting from the oxidation products that partly covered the electrode surface. In this section, the reproducibility was evaluated through SWV responses for 50 *µ*M AMX oxidation of 10 RGOnS electrodes, which were prepared under the same condition (Figure 7(a)). The calculated relative standard deviation (RSD) for ten measurements is 1.36%, illustrating the acceptable reproducibility of the electrode.

### 3.7. Calibration Curve  for AMX  Detection

Figure 7(b) shows the electrochemical response of RGOnS for AMX oxidation in the concentration range of 0.5 *µ*M to 80 *µ*M under optimized conditions. It is observed that the anodic peak signal (*I*_p_) varied linearly with the AMX concentration (C_AMX_). The linear regression equation was found as *I*_p_ (*µ*A) = 0.8684 + 0.2770 C_AMX_ (*µ*M) with the correlation coefficient of 0.9992.

The limit of detection (LOD) determined using following the equation LOD = 3*σ*/b was 0.193 *µ*M, where *σ* and *b* are the standard deviation and the slope of the regression line for the range 0.5–5 *µ*M, respectively. The calculated LOD was comparable with the ones reported in previous studies (Table 1) that confirmed the good sensitivity of the RGOnS electrode in AMX detection.

### 3.8. Determination of Amoxicillin in Different Water Samples

The practical performance of the RGOnS electrode was initially explored by the quantification of AMX in a drug capsule and in spiked water samples. The determination of AMX in each sample was performed 3 times to estimate the relative standard deviation (RSD). The recoveries were also calculated for determined concentrations of AMX as described in Table 2.

## 4. Conclusions

An efficient RGOnS sensor for AMX detection was successfully achieved from pristine graphite by the electrochemical method. Thanks to the formation of reduced graphite oxide nanosheet, the modified electrode showed a larger electrochemical active surface area, faster charge transfer, and more effective catalytic activity compared with those on the original electrode. These characteristics significantly contributed to the enhancement of the sensitivity of AMX detection with a low LOD of 0.193 µM. SWV responses on the RGOnS electrode show good linearity between peak current and AMX concentration and the noticeable reproducibility. The developed electrode was effectively applied in the quantification of AMX in real samples with good recoveries.

## Figures and Tables

**Table 1:** Comparison of the data obtained using electrochemical methods for the determination of amoxicillin.

Electrode	Method	Range (*μ*M)	LOD (*μ*M)	Ref.
MWCNTs/GCE	CV	0.6–8.0	0.2	[28]
AuNP-PdNP-ErGO	SWV	30 – 350	9	[30]
CPE	CA	0.195 – 14.6	0.088	[57]
PGA/3D-GE/GCE	SWV	2 – 60	0.118	[58]
PGA/GLU/GCE	SWV	2.0 – 25.0	0.92	[59]
Unmodified EPPG electrode	SWV	5 – 500	0.84	[27]
AuNPs/en-MWCNTs/SPE	AdSV	0.2 – 20	0.015	[29]
RGOnS	SWV	0.5 – 80	0.193	Present work

SWV : square-wave voltammetry, ^*∗*^DPV : differential pulse voltammetry, ^*∗*^CV : cyclic voltammetry,^*∗*^CA : chronoamperometric,^*∗*^AdSV : adsorptive stripping voltammetry, ^*∗*^CPE: carbon paste electrode, ^*∗*^GCE: glassy carbon electrode, ^*∗*^PGA: polyglutamic acid, ^*∗*^GLU: glutaraldehyde, ^*∗*^EPPG: edge plane pyrolytic graphite, ^*∗*^NP: nanoparticles, ^*∗*^SPE : screen printed electrode.

**Table 2:** Analytical results for the electrochemical determination of amoxicillin (*n* = 3) using the RGOnS electrode in different water samples.

Sample	C_AMX_ (µM)		Recovery (%)	RSD (%)
	Added conc.	Found conc.		
Distilled water	0	n.d.	—	—
	5	5.10	102.0	2.5
	10	10.26	102.6	3.1

Tap water	0	n.d.	—	—
	5	5.15	103.0	3.6
	10	10.15	101.5	4.0

West lake water	0	n.d.	—	—
	5	5.21	104.2	3.5
	10	10.68	106.8	4.8

Domestic wastewater	0	n.d	—	—
	5	4.72	94.36	4.7
	10	10.47	104.71	4.2

	Nominal value (mg/capsule)	Found value (mg/capsule)		
Drug	500	477	95.0	3.3

## Data Availability

The data used to support the findings of this study are included within the article.
